# Pregnant Women’s Intentions to Implement Safety Practices for Preventing Infant Injury: A Cross-Sectional Study

**DOI:** 10.3390/ijerph18010024

**Published:** 2020-12-22

**Authors:** Chikako Honda, Takashi Naruse, Noriko Yamamoto-Mitani

**Affiliations:** 1 Department of Community Health Nursing, School of Health Sciences and Nursing, Graduate School of Medicine, The University of Tokyo, Tokyo 113-0033, Japan; takanaruse-tky@umin.ac.jp; 2 Department of Gerontological Home Care and Long-Term Care Nursing/Palliative Care Nursing, School of Health Sciences and Nursing, Graduate School of Medicine, The University of Tokyo, Tokyo 113-0033, Japan; noriko-tky@g.ecc.u-tokyo.ac.jp

**Keywords:** infants, injury prevention, pregnancy, precaution adoption process model, suffocation, safety practice

## Abstract

Injury prevention education for pregnant women may be beneficial for infants’ safety. Currently, knowledge about the scope of an expectant mother’s intent to prevent injury is limited. The objective of this study was to determine pregnant women’s intentions to implement infant injury prevention strategies. From May to June 2017, a self-administered questionnaire based on the precaution adoption process model was distributed among pregnant women who participated in a parenting preparation class in a city, Tokyo. Pregnant women’s intentions to implement the following eight kinds of safety practices were measured: three practices regarding suffocation, two regarding falls, one safety practice for burns, one for accidental ingestion, and one for traffic accidents. Among 132 respondents (response rate: 83.5%; mean age: 33.4 years; mean gestational age: 29 weeks), the most common unawareness issue was “Make sure that there is no space between the mattress and bed frame” (68.2%), followed by “Use a firm mattress or futon” (38.5%) and “Keep soft objects away from the baby’s head in the baby’s sleep area” (31.8%); 58% or more women reported having already “decided to implement” the other five practices. Safety practices that pregnant women were mostly unaware of were for preventing suffocation, despite this being a leading cause of death in terms of unintentional infant injury. In comparison, the safety practices for falls, burns, and accidental ingestion were more known to pregnant women. The pregnant women’s intention to implement injury prevention for infants varied by safety practices. These findings could be used to improve the focus of antenatal education programs for the prevention of infant injury.

## 1. Introduction

Unintentional injury is a major cause of death in children. Injury prevention is currently perceived as a global public health challenge that requires a strategic response [1]. Incidence of injury in the home is higher among younger age groups; 93.4% of unintended injuries among infants (<12 months old) occur at home [2]. There is a high possibility that injuries in the home can be prevented by simply promoting the appropriate care to infant caregivers [3]. Thus, providing safety-related education for caregivers and expectant mothers could be an essential measure for preventing infant injuries.

In Japan, several laws recommend that health providers give new mothers or caregivers information about safety practices for infant/child injury prevention during their infant’s health checkups [4]. Currently, health providers such as public health nurses provide injury prevention information to caregivers during their child’s four-month health checkup [5]. Consequently, caregivers have very few opportunities to learn how to prevent injuries that could occur from birth until four months. After all, serious injuries and nonfatal injuries can occur even in early infancy [6,7]. It is necessary, therefore, to educate caregivers at an earlier stage than four months post-birth.

In this study, we focused on providing injury prevention education during the pregnancy period. Previous studies have shown that home visits throughout the pregnancy and postpartum period increase mothers’ safety-related knowledge and engagement in related behaviors [8] and reduce injury-related emergency room visits among children and the overall incidence of injuries [9]. In addition, some studies have reported that mothers prefer to receive such information during pregnancy [10].

Despite this, no previous studies have examined pregnant women’s intentions to implement safety practices for injury prevention to the best of our knowledge. The present study aimed to determine this specifically; its findings can help in the development of an educational program that provides appropriate safety-related information for women during pregnancy.

## 2. Materials and Methods 

### 2.1. Operational Definition

In this study, "infant" refers to children who are in early infancy, up to approximately four months after birth. 

### 2.2. Design and Sample

A cross-sectional study was conducted using a self-administered questionnaire in May–June 2017 at a public health center in the city of Tokyo metropolitan area. Participants were recruited from a parenting preparation class. In this city, parenting preparation classes were held three times a month, with around 20–30 attendees per session. Parenting preparation classes were open to residents who were pregnant with their first child and their partners, and these classes were advertised via the district’s website, etc. In 2016, the number of participants in the parenting preparation class was 1461, namely 56.3% of all registered pregnancies (2594), including those with a second or latter child in the city [11]. During the recruitment period, the parenting preparation class was held six times. 

At the beginning of each class, researchers explained the aim of this study and assured participants that the questionnaire was anonymous and voluntary. If participants agreed, they were asked to complete the questionnaire. Eligibility criteria for the study included women who (a) were pregnant with their first child, and (b) were able to understand and respond to the questionnaire in Japanese. Although the attendees in these classes included pregnant women and their partners or relatives, we asked only the pregnant women to respond to the questionnaire. Questionnaires were collected immediately after the classes.

The Ethics Review Board of our university approved this study (No. 11554-(1)) and was conducted by the Declaration of Helsinki.

### 2.3. Measures

We distributed questionnaires to measure the scope of pregnant women’s intentions to implement safety practices to prevent infant injury. Safety practice categories were developed through a literature review and a discussion with experts, and the prevention intentions to implement safety practices were measured based on the precaution adoption process model (PAPM) [12].

#### 2.3.1. Procedure of Determination of Safety Practices

First, we determined targeted injury types: suffocation, falls, burns, accidental ingestion, and traffic accidents. Suffocation and traffic accidents represented the highest incidences of accidental infant deaths in 2017 [13], while falls, burns, and accidental ingestion were responsible for the highest number of emergency room visits [14]. 

Next, we collected safety practices for preventing said injury types from various sources. The sources included guidelines by the American Academy of Pediatrics [15,16], the Tokyo Metropolitan Government Bureau of Social Welfare and Public Health [17], the Mother and Child book [18], and Japan Child and Family Research Institute [19]. Finally, we extracted items that overlapped (i.e., items that appeared in multiple sources). We asked six experts (a pediatrician, a pediatric emergency physician, a medical examiner, an injury prevention researcher, a public health nurse, and a midwife) to check the validity of these items. Through this process, we identified eight safety practices.

#### 2.3.2. Safety Practices

The following eight safety practices were determined to prevent the five targeted injuries. Three safety practices focused on preventing suffocation: (1) “use a firm mattress or futon”, (2) “keep soft objects away from the baby’s head in the baby’s sleep area”, and (3) “make sure that there is no space between the mattress and bed frame”. Item (1) “use a firm mattress or futon”, is presented in the questionnaire as “Use a firmer futon for a baby or a firmer (crib) mattress for a baby.” In Japan, infant-dedicated futons are sold in the market. It is common for parents and infants to sleep in the same room, with their appropriate infant-dedicated futon or crib next to the adults’ futon or bed.

Two safety practices targeted the prevention of falls: (1) “do not place your baby on high surfaces, such as sofas or tables” and (2) “keep crib sides raised whenever you leave your baby in the crib”. One safety practice was for preventing burns: “do not drink or carry hot liquids when holding your child”. 

One safety practice was for preventing accidental ingestion: “keep medicines, batteries, and small products in locked cabinets, or more than one meter above the floor”. 

One safety practice focused on preventing injury during traffic accidents: “correctly use child-restraint seats at all times the baby is in a vehicle”. 

Sudden infant death syndrome (SIDS) is another primary cause of infant deaths; however, this was excluded from the target items. This was because most of the risk factors for SIDS and suffocation are strikingly similar (i.e., use a firm sleep surface, keep soft objects and loose bedding away from the infant’s sleep area). The American Academy of Pediatrics is expanding its recommendations from being only SIDS-focused to focusing on means of creating a safe sleep environment that can reduce the risk of all sleep-related infant deaths, including SIDS [16]. In addition, a leaflet containing information for preventing sudden unexpected death in infancy, including SIDS (ensuring that the baby sleeps in the supine position, breastfeeding, avoidance of exposure to tobacco smoke), was distributed at the usual parenting preparation classes in this center.

#### 2.3.3. Precaution Adoption Process Model

**Figure 1:**
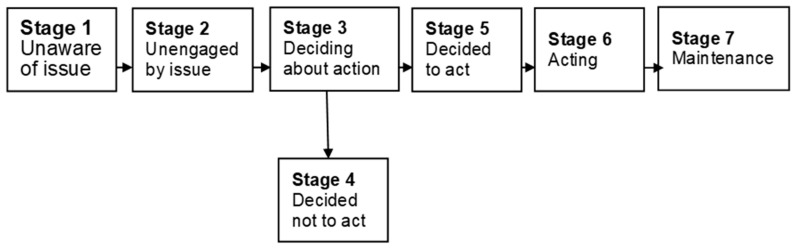
Stages of the Precaution Adoption Process Model. Note: Sourced from Weinstein, Sandman, and Blalock (2008).

We measured the mother’s intentions to implement each preventative safety practice using the PAPM [12], which is a stage-based behavior change model. These stages consist of: Stage 1 “unaware of issue” (i.e., do not know about the risk), Stage 2, “unengaged by issue” (aware of the risk, but have not considered adopting a preventive behavior); Stage 3, “deciding about implementing” (considering adopting the preventive behavior); Stage 4, “decided not to implement”; Stage 5, “decided to implement”, and Stage 6, maintenance (implementing the preventive behavior); see Figure 1 [20].

The PAPM suggests that people at different stages in the precaution adoption process behave in qualitatively different ways and that the interventions and information needed to move people closer to action vary across stages [12]. Responses to the questionnaire items were on a five-point scale ranging from “I do not know” to “I decided to implement”.

### 2.4. Statistical Analysis

Descriptive statistics (means and standard deviations (SDs) for continuous variables and the total numbers and percentages for categorical variables) were performed. SPSS version 24.0 (IBM, Armonk, NY, USA) for Windows was used for all statistical data analysis.

## 3. Results

### 3.1. Sample

We approached 158 pregnant women for participation in this research. Of these, 132 (response rate: 83.5%) agreed to participate and returned completed questionnaires. The mean age (range) of the respondents and their partners was 33.4 (26–50) years and 34.6 (25–52) years, respectively, and the mean gestational age (range) was 29 weeks (21–37).

### 3.2. Participants’ Intention to Implement Each Safety Practice

Table 1 shows, for each safety practice, the distribution of the implementation intention stages. Among all safety practices, the highest percentages for Stage 1 (unaware of issue) were “Use a firm mattress or futon” (38.5%), “Keep soft objects away from the baby’s head in the baby’s sleep area” (31.8%), and “Make sure that there is no space between the mattress and bed frame” (68.2%), all of which were the safety practices for preventing suffocation. 

The highest percentages for Stage 5 (decided to implement) were safety practices for all of the other target injuries. They were safety practices for preventing falls, burns, accidental ingestion, and traffic accidents. However, the second-highest percentage in each stage varied by safety practice. Among the falls and burns-related items, namely “Keep crib sides raised whenever you leave your baby in the crib” (58.9%) and “Do not drink or carry hot liquids when holding your child” (61.5%), the second-highest was Stage 1. Among the falls, accidental ingestion, and traffic accidents-related items, namely “Do not place your baby on high surfaces” (61.8%), “Keep medicines, batteries, and small products in locked cabinets, or on more than one meter above the floor” (63.4%), and “Correctly use child-restraint seats at all times the baby is in a vehicle” (75.4%), the second-highest was Stage 2 and 3.

## 4. Discussion

The analysis showed that most participants were at Stage 1 for safety practices preventing suffocation. In addition, the percentages of Stage 5 were lower compared to the safety practices for other target injuries. Despite the fact that suffocation accounts for nearly 70% of infant deaths [21], our findings suggest that pregnant women were not aware of and did not intend to implement safety practices for preventing suffocation. This indicates a need for a standardized program regarding suffocation prevention for expectant mothers and caregivers.

There are two possible reasons for this. First, in Japan, no proactive approach has yet been undertaken to raise awareness about safe sleeping environments during all periods of pregnancy and postpartum. In the United States, caregivers and healthcare providers are provided with education on safe sleep practices through the “Safe to Sleep©” campaign and guidelines from the American Academy of Pediatrics [16]. Second, health providers may not properly communicate the risks to caregivers because they find that it causes undue anxiety in the caregiver. In an interview with public health nurses and midwives, they reflected on the challenges of communicating the risks of suffocation to caregivers. In the PAPM structure, for people to adopt preventive behaviors, they need to be aware of the risks. It would be beneficial to incorporate the views of healthcare providers and pregnant women into the program development, not to put undue stress on caregivers but to make them aware of the risks.

For preventing falls, accidental ingestion, burns, and traffic accidents, the most common answers were Stage 5 (decided to implement; approximate 60%). Although many participants showed intention to implement safety practices, in reality, increasing safety practices has long been a challenge. In particular, falls are the most common cause of nonfatal injuries in infants [22]. Therefore, an injury prevention program should include identifying intention implementation gaps in safety practices and incorporating means of addressing these gaps. The latter would include education for live-in family members other than pregnant women in the program. Such content may help to link intentions of implementation in pregnancy to postpartum and to reduce the failure to implement safety practices by live-in family members.

On the other hand, the proportion of responses outside of Stage 5 varied across safety practices. This suggests that we should consider the circumstances and characteristics of each safety practice and reflect on the program’s content. For example, among the safety practices related to preventing falls, “Keep crib sides raised” and “Not place your baby on high surfaces such as sofas or tables”, the second-highest percentage of respondents was Stage 1 and Stage 2 and 3, respectively. The former practice of raising a crib side might be a difficult behavior to imagine in itself if a pregnant woman has never seen a baby crib. As for the latter practice, pregnant women might not understand that the baby may move and fall even if the baby cannot turn over. It is essential to include information in the program that allows pregnant women to imagine the situation in which their babies are at risk and understand why safety practices are needed.

Regarding safety practices for preventing traffic accidents, the awareness of the risk and the intention to implement was higher than other safety practices. One of the reasons for this is that the use of child restraint seats is compulsory by law in Japan. In this sample, only half of the respondents owned a car. The municipalities of Tokyo have the lowest rate of car ownership per household in Japan (43.1%) [23]. At a public health center within a community with low car utilization rates, we can prioritize whether we should include safety practices for traffic accidents in our programs. At the same time, it may be necessary to add to the program that families must use taxis equipped with child seats when returning home from the hospital

This study has several limitations. The sample size for this survey was relatively small and drawn from parents resident in an urban area of Japan who attended parenting classes. Therefore, study results were not generalizable to all Japanese parents living in numerous geographical regions of Japan who have different sociodemographic characteristics and varied access to antenatal education and parenting support. Educational status was also not measured; however, it is a demographic characteristic associated with risk factors for child injuries. Based on previous studies conducted in this region [24], it is likely that participant parents held educational levels higher than the national average. Therefore, the deficits identified are likely to be an underestimate of parenting knowledge of safety practices generally.

Despite these limitations, this study is the first to examine pregnant women’s intentions to implement safety practices for infants.

## 5. Conclusions

Pregnant women’s intention to implement injury prevention for infants differs across safety practices. Safety practices for preventing suffocation were the least known despite this being a leading cause of death in terms of unintentional infant injury. The present results should be utilized as a reference to develop an appropriate program to improve the implementation rate of safety practices for the prevention of infant injury.

## Figures and Tables

**Table 1:** Participants’ Intention to Implement Safety Practices to Prevent Infant Injuries (n = 132).

	Safety Practice	Intend to Implement (n/%)		Do not Intend to Implement(n/%)					
		<Stage 5> Decided to Implement		<Stage 1> Unaware of Issue		<Stage 2> Unengaged by Issue<Stage 3> Deciding about Implementing		<Stage 4> Decided not to Implement	
Suffocation	1. Use a firm mattress or futon	55	(42.3)	50	(38.5)	24	(18.4)	1	(0.8)
	2. Keep soft objects away from the baby’s head in the baby’s sleep area	47	(36.4)	41	(31.8)	37	(28.7)	4	(3.1)
	3. Make sure that there is no space between the mattress and bed frame	14	(10.6)	90	(68.2)	2	(1.5)	26	(19.7)
Fall	4. Do not place your baby on high surfaces, such as sofas or tables	81	(61.8)	10	(7.6)	39	(29.8)	1	(0.8)
	5. Keep crib sides raised whenever you leave your baby in the crib	73	(58.9)	30	(24.2)	20	(16.1)	1	(0.8)
Burn/Scald	6. Do not drink or carry hot liquids when holding your child.	80	(61.5)	27	(20.8)	16	(12.3)	7	(5.4)
Accidental ingestion	7. Keep medicines, batteries, and small products in locked cabinets, or on more than one meter above the floor	83	(63.4)	19	(14.5)	25	(19.0)	4	(3.1)
Traffic accident	8. Correctly use child-restraint seats at all times the baby is in a vehicle *	49	(75.4)	1	(1.5)	15	(23.1)	0	(0.0)

* Excluding 67 people who did not own a car.
